# The Balance of Weak and Strong Interactions in Genetic Networks

**DOI:** 10.1371/journal.pone.0014598

**Published:** 2011-02-10

**Authors:** Juan F. Poyatos

**Affiliations:** 1 Logic of Genomic Systems Laboratory, Spanish National Biotechnology Centre, Consejo Superior de Investigaciones Cientficas (CSIC), Madrid, Spain; 2 Department of Biology, Centre for Ecological and Evolutionary Synthesis (CEES), University of Oslo, Oslo, Norway; University of Glasgow, United Kingdom

## Abstract

Genetic interactions are being quantitatively characterized in a comprehensive way in several model organisms. These data are then globally represented in terms of genetic networks. How are interaction strengths distributed in these networks? And what type of functional organization of the underlying genomic systems is revealed by such distribution patterns? Here, I found that weak interactions are important for the structure of genetic buffering between signaling pathways in *Caenorhabditis elegans*, and that the strength of the association between two genes correlates with the number of common interactors they exhibit. I also determined that this network includes genetic cascades balancing weak and strong links, and that its hubs act as particularly strong genetic modifiers; both patterns also identified in *Saccharomyces cerevisae* networks. In yeast, I further showed a relation, although weak, between interaction strengths and some phenotypic/evolutionary features of the corresponding target genes. Overall, this work demonstrates a non-random organization of interaction strengths in genetic networks, a feature common to other complex networks, and that could reflect in this context how genetic variation is eventually influencing the phenotype.

## Introduction

The study of biological networks is beginning to expose how the combination of basic characteristic elements brings about system-level behaviors. These networks represent in many cases the integration of processes very well delineated molecularly, such as transcription [1], metabolism [2], or protein-protein interaction [3]; processes (and networks) that should ultimately be aggregated to properly describe cellular physiology [4].

A possible exception to this view corresponds to the specific case of genetic interaction networks [5]. These networks are not so much linked to a particular molecular process, but to the conceptual idea of the genotype-to-phenotype map, and the dependence of such map on the associated genetic background. Both notions were initially raised in the early days of genetics, when a number of studies started to approach the issue of how gene interactions could influence the function and evolution of genetic systems [6]. Such gene interactions were broadly termed epistasis, and referred largely to the fact that the contribution of a single locus to the genotype-to-phenotype map could depend on the genotype at another genomic location [7].

**Figure 1:**
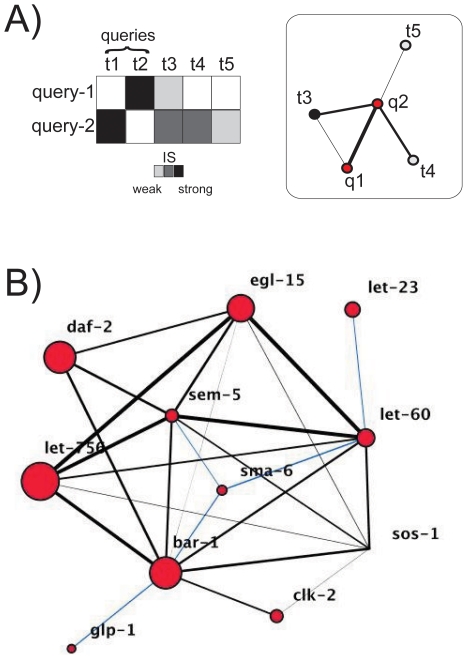
Genetic interaction networks. A) Genetic interactions of different strengths between query and target genes constitutes the genetic network (red nodes represent query genes (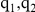); white/black nodes represent target genes interacting with one (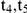) or more than one (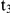) query. B) The *C. elegans* query network –constituted by the interactions between query genes only– represents the functional associations between different signaling pathways. IS is represented by the width of the edges, while the number of interactions with target genes other than queries (target-connectivity) is qualitatively described by node size. Those interactions of relatively weak strength that appeared most important to maintain the structure of functional linkages among pathways (network as a single-connected component, see main text) are highlighted in blue.

The analysis of genetic interactions, and its systematic mapping to establish genetic networks, benefited enormously from the application of newly developed high-throughput experimental technologies. These tools are based on the possibility of generating collections of single gene mutants –both in unicellular [8], [9], [10], [11], [12] and multicellular [13], [14], [15] model organisms, and also in mammalian systems [16], [17] – that are then queried against a second large set of target gene mutations (Figure 1A and Box 1). Genetic interactions are thus defined for those cases in which the growth of the double mutant is different to its (expected) growth in the absence of any relationship (expected growth is usually quantified by the multiplicative effect of the single mutations (see [18], [19] and Materials and Methods).

What type of biological questions can we analyze with the use of genetic networks? One could generally consider three classes. The most direct question should be what actually represents a genetic interaction in molecular terms, e.g., [20]. Answers to this question were proposed already with data generated in the first systematic studies but they could only be of limited scope, as the type of interactions being measured (initial studies were only linked to a particular case of negative genetic interaction termed synthetic lethality, see Materials and Methods). Synthetic lethal interactions were hence proposed to represent the functional dependence of two genes acting in parallel pathways, while the number of interactions exhibited by a particular gene helped to reveal its position within a pathway [21]. Recent experiments are now able to quantify a wider range of interactions, from negative to positive, and consequently more clear patterns are expected to emerge [22], [23], [24], [25].

A second group of questions should be related to the integration of bioprocesses, i.e., the functional cartography of cellular pleiotropy [12]. Patterns of target interactions for each query gene can be considered as valid phenotypic signatures and thus clustered –similar patterns revealing functional association among the corresponding query genes. This use of genetic interactions as a tool to uncover function improved again with the use of more quantitative data, such as that obtained with the use of dSLAM (diploid synthetic lethality analysis with microarrays [10]), GIM (genetic interaction mapping [11]) and SGA (synthetic genetic array [12]) techniques.

Finally, a third set of questions could be asking about the structural properties of genetic networks, and how these properties can reveal organizing principles of the underlying biomolecular systems, e.g., [8], [14]. Two main structural features are noticeable. First, genetic networks present a number of genes with large connectivity, or hubs, particularly enriched with chromating remodeling functions [13], [26]. This presents such genes as modifiers of many diverse biological processes with two seemingly contradictory consequences; their presence buffers biological systems from a large number of gene mutations, i.e., it limits change, while their absence could unveil otherwise hidden variation [26], i.e., it promotes change. A second interesting property is the poor conservation of genetic interactions in different organisms unlike other biomolecular networks, although the exact level of conservation is still uncertain [27], [28], [29].

This work belongs to the last class of questions. I specifically ask about the distribution of interaction strengths (ISs) in genetic networks. By analyzing several network features I observe a non-random association between these attributes and ISs. I then discuss the consequences of these patterns for the underlying biomolecular systems.

## Results

### Weak interactions are important to preserve the structure of functional linkages among pathways

I first analyzed the query network linked to a recent systematic study in the nematode *Caenorhabditis elegans* (Materials and Methods and Figure 1B). This network is constituted by the genetic interactions uncovered between query genes, a set of genes associated to six fundamental signaling pathways in metazoans (the EGF, FGF, Notch, insulin, Wnt and TGF-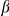 pathways [14]), that are mutated in human diseases [13]. The presence of genetic interactions between these genes indicates that components of alternative pathways could be functionally buffering each other (one gene of a given pathway rescuing the function of a different pathway in which its associated query gene is deleted; note here that *clk2*, specifically related to DNA-damage response, could be broadly considered a signal transduction gene). Furthermore, the fact that the query network constitutes a single-connected component could indicate the physiological relevance of a full association among all pathways. Which gene is then more central to maintain this network structure? I knocked out each query gene (by zeroing its associated connections) in the network independently, and measured the average shortest distance between nodes as a proxy of the (mutant) network functional connectivity; with larger distances indicating higher pathway isolation. Intuitively, the more central the deleted node was (as denoted by its query network connectivity), the less functionally connected the mutant query network became (larger average shortest distance, Spearman 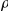 = 0.7, 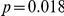, most central nodes were *bar-1*, *let-60*, and *sem-5*). Note here that centrality was a predictor of the node mean IS in the query network (edge widths in Fig. 1B, 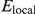 = 0.64, 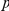 = 0.035), but not of the number of interactions of the query nodes with target genes (node size in Fig. 1B; 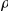 = 0.33, 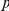 = 0.31).

**Figure 2:**
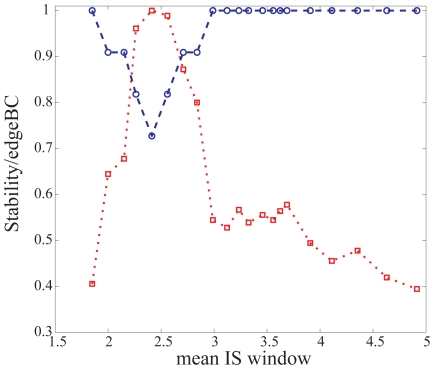
Deletion of five interactions (edges) in a sliding windows analysis with increasing IS. Relatively weak edges produced the largest change on network structure (as measured by the size of the largest connected component normalized to the maximum, blue circles). These weak edges were the ones most frequently connecting pathways (largest edge betweeness centrality, eBC, normalized to maximum, red squares). Dashed and dotted lines to help visualization.

What about query-query interactions? Which ones could be more important to sustain full connectivity? One might *a priori* expect a relationship between query-query links with strong ISs and their effect on connectivity when deleted. However, this was not the case. I deleted five edges in a sliding window analysis of increasing IS (this was the average number of deleted edges when a single gene was knocked out). I found that edges of relatively weak IS were instead the most relevant to network structural stability (size of the largest connected component, Figure 2). These edges were the ones most frequently connecting pathways (this is quantified in graph theory by the average edge betweeness centrality, eBC, i.e., the number of times that a particular edge takes part in the shortest path between two nodes in a graph). Indeed, there exists an anticorrelation between average eBC and average IS (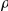 = −0.62, 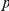 = 0.0034), with the former evidently related to stability (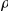 = −0.84, 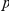 = 2.5 10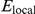). Multiple gene knockouts involved in weak (double mutant) interactions could thus have a strong effect [22]. Overall, these results manifest that weak connections are important to keep the structure of functional linkages among signaling pathways.

### Genetic interaction patterns depend on interaction strength

**Figure 3:**
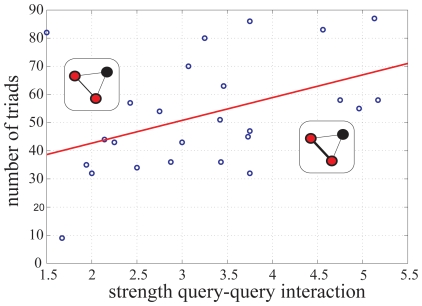
The number of triads established by pairs of interacting query genes correlates with the strength of this interaction. Dots represent the number of triads for each interacting query pair, with the red line representing the regression curve with 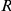 = 0.44, 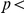
0.03 (IS represented by edge width; query and target genes as red and black circles, respectively). Genes involved in the strongest ISs are part of the fibroblast growth factor pathway: ( *egl-15*, *let-60*}, (*egl-15*, *let-756*}, ( *sem-5*, *let-60*}, and ( *sem-5*, *let-756*}, see also Fig. 1B.

I then analyzed the global patterns of interactions between query and target genes. These interactions act as truly phenotypic signatures to identify functionally related genes by means, for instance, of two-dimensional clustering of query and target genes with similar profiles [8], [14], [11], [12]. Here, I present a somehow complementary study. I examined whether the structure of the query network itself could be determining the patterns of interactions with target genes. First, I considered pairs of interacting query genes and asked to what extent these pairs showed a stronger trend to interact with the same target genes, as compared to pairs of non-interacting query genes (note that by target genes I considered only those targets which were not query genes too, see Figure 1A). Interacting query genes showed a stronger tendency to act with the same target genes than expected by chance (score 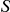 defined as the number of common targets –of a total of 450– between query pairs; 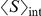 = 53, 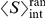 = 44.3, 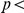
0.001, non-parametric permutation test in which I took random sets of query pairs and then measured 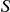, 10000 times), while the opposite was found for non-interacting pairs (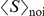 = 37, less number of interactions than expected by chance 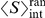 = 44.3, 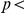
0.001, permutation test, 10000 times). In addition, the number of common interactions, i.e., number of triads, established by pairs of interacting query genes correlated with the IS of the interaction (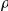 = 0.48, 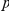 = 0.016, Figure 3).

Moreover, I computed the mean IS of the target genes that interacted exclusively with query genes (query-connectivity  = 1), and compared it with the mean IS of those target genes interacting inclusively with query genes (query-connectivity 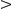
1). Inclusive interactions showed a higher mean IS than expected by chance (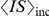 = 3.18, 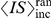 = 2.94, 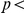
0.0001, where I randomized ISs of target-query interactions 10000 times keeping network topology), while exclusive interactions showed a lower mean IS (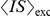 = 2.67, 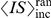 = 2.94, lower than expected with 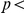
0.0001, randomization as before).

### The *C. elegans* genetic network does not exhibit strong genetic cascades

To further understand the distribution of ISs in the *C. elegans*' network, I made use of a quantitative framework recently proposed in studies of weighted complex networks, the network efficiency [30], [31]. To this aim, I first introduced a notion of “functional distance” by reinterpreting the strength of the genetic interaction between two genes. For every genetic interaction between two genes 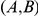, I defined this distance as the inverse of the IS, i.e., 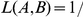
IS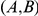. “Close” genes in this metric reflect thus strong (negative) epistasis, which intuitively suggests a proximate functional relationship [5].

I then considered the concept of efficiency. Imagine that one measures the weighted shortest path between every pair of genes in the network, 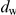. By this I mean the path connecting two genes with the smallest sum of edge distances 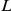 (from all the possible paths connecting them). Two genes are efficiently connected if 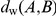 is small. One can take now the average of all weighted shortest paths, or rather the average of the inverse, 1/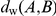, to determine network efficiency. Small shortest paths between genes imply that their inverse is large and that the network efficiency is equivalently large. Finally, efficiency can be normalized by its maximum possible value that could be obtained if all genes were connected in the network (the *ideal* network, Materials and Methods). Following these definitions, I obtained a global efficiency of the *C. elegans* genetic network of 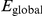 = 0.21, i.e., 21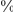 of the ideal network. This value was always less than that obtained in networks with same topology but randomized ISs (mean value of 1000 randomizations 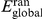 = 0.23, 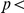
0.001).

Efficiency, in its standard application, broadly measures how well information propagates over a network [31], with high efficiency implying the presence of small shortest paths between nodes. In the case of a genetic network, maximal efficiency would imply that genes usually take part of genetic cascades of the type 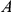
–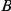
–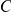
–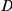 constituted by pairwise interactions with strong IS. A network with low efficiency, like the one observed here, suggests otherwise absence of these cascades. While high global efficiency is considered a positive attribute in most networks –so that global communication in the network is optimal [30], [31] –, high global efficiency might be denoting a disturbing structural property in the case of genetic networks, as it indicates that a single gene inactivation leads to a number of particularly strong deleterious cascade effects.

Specific patterns of ISs could be additionally identified with the network local efficiency. This score represents how robust is the connectivity between first neighbors of a chosen node, when this node is removed, i.e., how fault tolerant is the network to node removal (Materials and Methods) [30], [31]. In the context of genetic networks, local efficiency denotes how many genes linked to a specific genetic modifier are also linked to alternative modifiers. The observed local efficiency 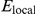 = 0.278 was bigger than the random value, but this difference was not statistically significant (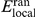 = 0.273, 1000 randomizations as before, 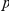 = 0.25). Interestingly, when I computed the local efficiency of query genes only, I did observe that the restricted local efficiency was significantly larger than expected by chance (observed 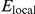 = 0.184, random 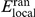 = 0.169 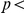
0.001). This suggests that, on average, several query genes could act as modifiers of similar target genes since the removal of a single query changes the connectivity of its first neighbors less than what is randomly expected (the network structure is particularly fault tolerant).

**Figure 4:**
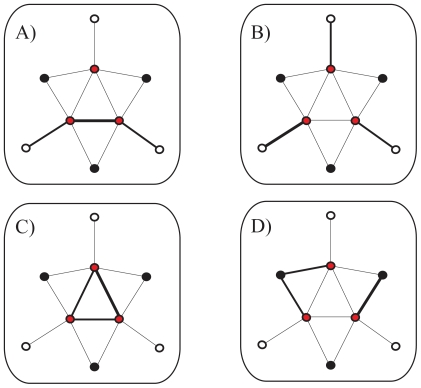
Global and local efficiency in genetic networks. In this toy network query genes, genes with inclusive target-query interactions and genes with exclusive target-query interactions are shown as red, black and white nodes, respectively. IS is represented by the edge width. A) maximal global efficiency correspond to cascades of strong IS (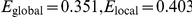), B) minimal local efficiency corresponds to exclusive interactions of strong IS (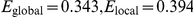), C) maximal local efficiency corresponds to query interactions of strong IS (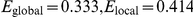); this also corresponds to maximal local efficiency of query genes only, D) minimal global efficiency correspond to inclusive interactions of strong IS (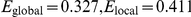). The *C. elegans* and *S. cerevisiae* networks would be a mixture of network models C) and D), see main text for details.

One could understand the previous patterns by discussing four limiting situations in a toy network (Figure 4). First, one could imagine a network in which the ISs of some query-query and exclusive target-query interactions are usually strong, while the inclusive target-query ones are weak (network A, Fig. 4A). This implies the presence of short-distance cascades crossing the graph and hence maximal global efficiency. An alternative IS distribution could correspond to strong exclusive target-query interactions, with the rest being weak (network B, Fig. 4B). This would generally minimize local efficiency as the network query genes at the core are at very large distances. The opposite situation in which only query-query interactions are strong maximizes local efficiency (network C, Fig. 4C). Finally, one could consider a network in which strong ISs are mostly distributed on inclusive target-query interactions, which minimizes global efficiency (network D, Fig. 4D). The *C. elegans* network showed minimum global efficiency which would indicate that it corresponds to the last model, i.e., a situation with low global efficiency and weak exclusive interactions (recall that exclusive interactions showed a weaker mean IS than expected by chance), in combination to network C, which also reflects the maximal local efficiently observed when knocking out query genes.

### Genetic hubs act as especially strong modifiers

**Figure 5:**
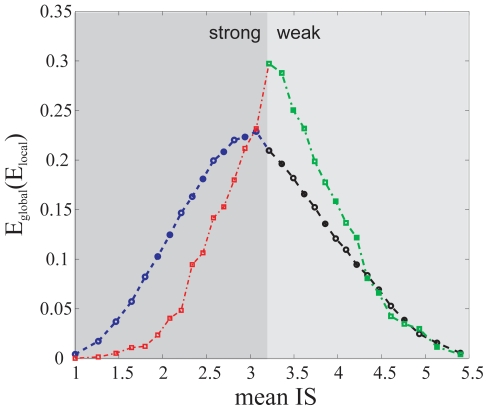
Change of global and local efficiency as a function of mean IS in mutated network. Mutated networks were obtained after increasingly deleting interactions up to a core network. Blue, change in 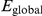 when increasingly deleting strong interactions. Black, change in 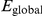 when increasingly deleting weak interactions. Red, change in 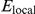 when increasingly deleting strong interactions. Green, change in 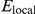 when increasingly deleting weak interactions (lines between points to help visualization; dark gray shading denotes deletion starting from strong edges while light gray denotes deletion starting from weak edges).

Could we specifically characterize the role of strong interactions in the architecture of these networks? I used again the previous network measures to consider the two following scenarios. In the first one, I deleted an increasing number of edges, based on its strength, until I reached a core network. I obtained a contrasting behavior depending on whether deletion started from weak or strong edges (Figure 5). 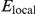 decayed faster when deleting strong interactions because these interactions are those contributing more to local fault tolerancy. Genes with high connectivity (query genes) provide alternative routes to connect target nodes (i.e., mutations on these target nodes could be buffered by different queries). As these genes are involved, on average, in interactions with strong IS, deletion of strong links reduces these alternative routes, i.e., the network local fault tolerance. Weak interactions, on the other hand, are more specific to single query genes contributing less to the previous pattern, as indicated by the slower decay of the network local robustness, i.e., 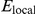.

In comparison, 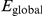 decayed faster when deleting weak interactions (Fig. 5). This is due to the fact that more genes get disconnected (as weak interactions are commonly related to exclusive query-target interactions), not contributing to the global efficiency; indeed, the size of the largest connected component decreases considerably when deleting weak interactions (data not shown).

In a second scenario, I knocked out those target genes with the largest query-connectivity (top 25 genetic hubs [13]), and quantified the global efficiency of the mutated network. The mutated network decreased in efficiency (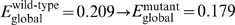) and this decrease was larger than expected by chance (mean random 
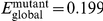
, 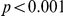, considering 1000 random networks in which ISs were randomly assigned). In sum, these analyses emphasize the interconnection among strong ISs, inclusive interactions, and genetic hubs.

### Interactions strengths are also not randomly distributed in *S. cerevisiae*

Could one find the previous patterns in other genetic networks? This might not necessarily be the case as genetic interactions do not appear to be conserved in different organisms [27] (but see [28], [29]). To investigate this, I first used a genetic network associated to the process of mRNA decapping in the yeast *Saccharomyces cerevisae* [11] (Materials and Methods). I obtained again that 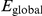 is smaller than expected by chance (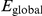 = 0.061, 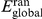 = 0.066, 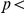
0.001, randomizing ISs, 1000 times). In this case, local efficiency was significantly larger than what it was randomly observed (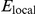 = 0.11, 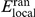 = 0.098, 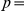
0.035, randomizations as before). Moreover, knocking out of genetic hubs also led to a larger decrease in global efficiency than expected (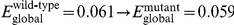, mean random 
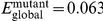
, 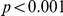), confirming the picture of hubs as particularly strong genetic modifiers.

These patterns were also observed in a recent, and much larger, yeast dataset [12]. Global efficiency was again lower than expected by chance, with local efficiency being larger (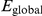 = 0.076, 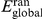 = 0.08 and 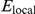 = 0.037, 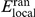 = 0.031; both cases with 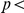
0.002, randomizing ISs 500 times). Additionally, interacting query genes exhibited a larger number of common target interactors, a pattern that depended on IS (data not shown); both results similar to those found in the *C. elegans* network.

Finally, I used this second dataset [12] to analyze the potential association between ISs and the corresponding physiological and evolutionary attributes of the genes involved. Specifically, I computed the correlation between query-target ISs and a number of attributes of the target genes (e.g., multi-functionality, expression level, etc, Table 1 and Materials and Methods). I compared these scores to those obtained after random permutation of each attribute value within each genetic connectivity class (number of genetic interactions) of the target gene. This protocol is aimed to control for the already known signal between target gene connectivity and the physiological/evolutionary attributes considered, as shown in [12]. Most features showed the same tendency observed with connectivity when IS was further considered. For instance, pleiotropy (as measured by the multi-functionality and phenotypic capacity attributes) and conservation (as measured by copy number volatility and 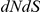) correlated positively and negatively, respectively, with connectivity and they also did it with IS –when genetic connectivity is controlled for– as compared to a null. The strength of these associations was however small.

**Table 1:** Correlation between query-target IS and different attributes of the corresponding target gene.

	*Physiological and evolutionary properties*									
	Single mutant	Multi-	Phenotypic	Chemical-genetic	PPI	Protein	Expression	Yeast	Copy number	
	fitness defect	functionality	capacitance	degree	degree	disorder	level	conservation	volatility	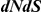
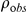	0.115	0.048	0.052	0.04	0.042	0.004	0.022	0.018	−0.058	−0.03
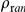	0.127	0.04	0.044	0.037	0.045	0.006	0.03	0.02	−0.04	−0.024
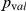	0.0001	0.0088	0.0029	ns.	ns.	ns.	0.009	ns.	0.0001	0.0365

First row is the observed value. Second row is the mean value observed in 10000 permutations of the attributes, controlling for target connectivity (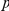-values of these tests shown in third row; ns.: statistically non-significant).

## Discussion

Genetic networks are the result of a systematic strategy to map the functional associations characterizing a biological system by means of perturbations (Fig. 1). How are such functional associations ultimately identified? One approach is to link each genetic interaction to its molecular underpinnings, with the goal of determining general patterns between classes of interactions and what they represent, e.g., [20], [21], [24]. A complementary strategy is to search for organizing principles in the genetic network itself, and then analyze the potential implications of these principles in the underlying biological system, e.g., [32], [25]. I followed here this second approach by focusing on understanding the distribution of (negative) interaction strengths in genetic networks.

Using data from a *C. elegans* genetic network linked to a set of conserved metazoan signaling pathways, I obtained two main patterns associated to the strength of these interactions. I observed first that weak interactions are important to maintain the structure of buffering linkages among pathways (Fig. 2, these weak interactions involved genes, such as *glp-1* or *sma-6*, of different pathways). I also found that the presence/absence of a genetic interaction between two signaling genes influence the number of common (target) interactors they exhibit. This confirms the view that correlated interaction profiles between two genes suggest shared function [8], [14], [11], [12] –in this case reflected in the presence/absence of a genetic interaction between such two genes. Indeed, the strength of the genetic interaction acted as a significant predictor of the number of common interactors the corresponding signaling genes exhibit (Fig. 3, those pairwise interactions with the strongest IS –and thus with the largest number of common target interactors– involved query genes which were orthologs of members of the fibroblast growth factor pathway).

I considered two additional genetic networks characterized in yeast, together with the nematode data, to further study the arrangement of ISs (the molecular techniques to generate these networks are considerable different, see Materials and Methods, but they are ultimately produced with the same query-target approach [5]). Adopting a framework from complex network theory (network efficiency [30], [31]), I first observed that genetic networks did not generally show cascades constituted by strong pairwise interactions. This indicates that in gene cascades of the type 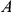
–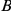
–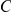
–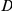, the IS between 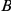
–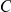 (both query genes) is loosely linked, on average, to the IS of the interactions 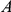
–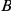 and 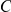
–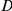 (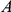, 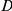 being exclusive target genes, Fig. 4). The strength of the interaction between two genes can act then as a predictor of the number of common genetic interactors, but not so much of their interaction strengths. Moreover, this balance of interaction strengths could reflect and underlying biological organization that limits the propagation of deleterious effects and that resembles the monochromatic structure of interactions in metabolism (in which different groups of genes exhibit opposite types of epistasis in their intra- or inter-group relations [32]).

I also found that weak interactions are important for full network connectivity (as they are linked to exclusive query-target links) while strong interactions are relevant for local fault tolerance to genetic mutations (being linked to inclusive query-target interactions, Figs. 4,5). In addition, ISs of the most inclusive target genes (hub target genes interacting with many queries) showed a distinct distribution of strong genetic interactions. This distribution presents these genes –enriched in various cellular processes [26] – as particularly strong phenotypic modifiers, i.e., their absence revealing a large number of hidden mutations causing particularly strong changes in growth [13]. In yeast, I also observed a weak association between IS and some phenotypic/evolutionary attributes of the target genes involved (Table 1).

In sum, a non-random balance of weak and strong interactions in genetic networks clearly emerges from this analysis –a balance that we might well feel a nontrivial common property of complex systems [33], as it is characteristic of other networks [32], [34]–[36]. However, the implications of this IS distribution, and of other patterns found in related works, for the organization of the underlying biological systems appears sometimes obscure. I believe this is due to three causes. First, it can be a consequence of technical limitations derived from the the biased sampling of query and target genes, with the number of genes acting as queries being always considerably smaller than those acting as targets. In this sense, the network constituted by the query genes associated to [12] could be the best current picture of a large network in which all potential genetic interactions between the constituent genes were scored. Notably, both the local and global patterns uncovered by the use of the network efficiency framework were also observed in this yeast query network. Second, it could also be caused by the different quantitative definitions used for genetic interaction, e.g., [18], [19]. Finally, and most importantly, it can be originated by the intrinsic difficulty to map patterns observed in a conceptual network, constructed on a specific perturbation strategy of a system, to the underlying structural organization and function of that very same system. This mapping might not even be stable [37].

Efforts to understand these networks, further generalizations of perturbation approaches, e.g., [22], [25], and integration with forward genetic strategies (e.g., genome-wide association studies [38]) are nevertheless necessary if we are to understand how genetic interactions influence the evolution of biological systems, and, from a biomedical side, how these interactions contribute to relevant human quantitative traits.

## Materials and Methods

### *Caenorhabditis elegans* SGI genetic network

Data from a systematic genetic interaction (SGI) analysis by Byrne *et al* [14]. Query genes are hypomorphic mutants, with reduced but not eliminated function, of genes corresponding to signaling pathways in metazoans. Hundreds of target genes were inactivated in each query-gene background by using RNA interference techniques, see also [13]. The most robust network consists of 1246 interactions among 461 genes. The distribution of ISs in the SGI network hardly shows alleviating interactions (as compared to the whole interaction dataset, i.e., Fig. 3 in [14]). When assembling the interaction network, I found several cases of pairwise interactions with two different associated IS. In these cases, I took the mean (this implied that the only two alleviating interactions in the SGI dataset became positive).

### *Saccharomyces cerevisiae* GIM genetic network

Data from a genetic interaction mapping (GIM) by Decourty *et al* [11]. In this study 41 different query mutations of genes involved in several RNA metabolism pathways (Table S2 in [11]) were tested against a collection of 3812 target mutations giving rise to approximately 140.000 double mutant deletion strains. Only 1095 deletion strains of the collections gave a significant relative score and that is the data I analyzed (in those cases where the same query genes were involved in independent screens I took the mean relative growth value). To compare with the *C. elegans* network I considered only negative relative growth scores (note that this is a conservative subset of the negative genetic interactions) and took the absolute value so that strong interactions are the ones with the largest value. The resulting GIM network consists of 16838 interactions among 1106 genes.

### *Saccharomyces cerevisiae* SGA genetic network

Data from a recent genetic synthetic genetic array (SGA) study by Costanzo *et al* [12]. 1712 query genes, selected randomly with respect to function, were screened against 3885 target genes to give approximately 170.000 interactions. I considered a filtered data set at a defined confidence threshold for my analyses. To compare with the other analyses I only considered negative interactions (and took the absolute value so that strong interactions are the one with the largest value) to obtain a network with 108414 interactions and 4434 nodes.

### Defining genetic interactions

Negative interactions correspond to a more severe fitness defect in the double mutant than expected from the fitness of single mutants (such expected fitness can be defined in different ways, see [18], [19] and below). They are also termed enhancing, aggravating or synergistic interactions. A limiting case of negative interaction where double mutants are not viable is termed synthetic interaction; the first systematic studies characterized this class [8]. Positive interactions correspond to those cases where the double mutant fitness is greater than expected from the single mutant values. They are also termed alleviating interactions. See [5] for more details and references.

### Defining ISs

To quantify ISs Byrne *et al* [14] estimated progeny in double mutants and controls (query RNAi in wild-type background, and the control vector in the hypomorph background). ISs measured average growth difference between the double mutant and the control populations. This can be seen to represent a conservative estimate of the possible interactions obtained following a multiplicative model of expected fitness (see additional data file 5 in [14] for details). Costanzo *et al* [12] quantified ISs by estimating fitness effect directly from double mutants colony size and then contrasting this value with the expected multiplicative effect of combining the two corresponding single mutant scores. Finally, Decourty *et al* [11] ISs were obtained by comparing the differential enrichment of double mutants growing in competitive culture with two reference controls (using barcode microarrays). Reference controls included each target mutation in one/two backgrounds of neutral control deletions. This approach is similar to the dSLAM [10] technique, claimed to be using a minimum definition of expected fitness (two mutants are independent if the double mutant has the same fitness that the less-fit single mutant). While definitions of genetic interactions can be relevant, e.g., some could be better than others to identify functional relationships [18], the use of multiple definitions may still be valid to reveal complementary biological properties [19]. The analysis of this latter data suggests that different definitions could also help identifying common organizing principles of their corresponding genetic networks.

### Defining Efficiency

Efficiency was recently introduced as a quantitative measure to study information transfer in weighted networks [30], [31]. The efficiency between two nodes, 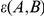, is given by the inverse of the corresponding weighted shortest path length (the smallest sum of distances throughout all the possible paths in the network from 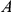 to 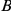), i.e., 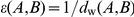. The average efficiency of a network, or graph 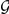, with 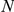 nodes is given by
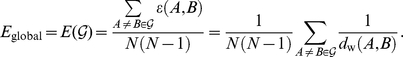



To obtain a normalized efficiency the previous score is divided by that of the ideal graph, i.e., the network with all possible edges (and thus information transfer is in the most efficient way). In the ideal genetic network, I gave the minimal characteristic distance to any two nodes not connected. To those cases where the direct pairwise interaction between two genes had a larger distance value that the one linked to undirected pathways, I assigned the lowest value of the two; these choices lead to maximal efficiency. Finally, local properties of the network can be evaluated by measuring the efficiency associated to each gene 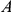, i.e.,
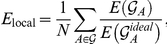



with 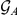 (and 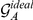) being the sub-network constituted by all the genetic interactions associated to gene 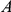 (and its corresponding ideal genetic sub-network). Local efficiency quantifies how much deterioration in the connectivity between the neighbors of 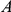 will occur when 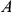 is removed, i.e., how much the system is fault tolerant.

### Genetic and evolutionary properties

I considered the following features in Table 1 (see [12] for details): 1) Single mutant fitness defect: 1-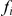, with 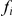 being the single mutant fitness defect derived from mutant colony size data, 2) multi-functionality: total number of annotations across a set of functionally distinct GO terms, 3) phenotypic capacitance: the number of quantitatively different morphological phenotypes linked to a specific gene, 4) chemical-genetic degree: sensitivity to a library of drugs as well as a variety of experimental conditions, 5) PPI degree: total number of interactions in the union of four high-throughput physical interaction datasets, 6) protein disorder: the percent of unstructured residues, 7) expression level: average number of mRNA copies of each transcript per cell, 8) yeast conservation: number of species that possess an ortholog of a given gene, when considering 23 different species of Ascomycota fungi, 9) volatility: frequency of gain (including duplication) or loss events across the 23 species before, 10) 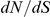: 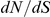 ratio for *S. cerevisiae* in comparison to the *sensu strictu* yeast species *S. paradoxus, S. bayanus and S. mikatae*.
